# AI Perceptions, Professional Identity, and AI-Supported Clinical Decisions Among Medical Students and Clinicians: Cross-Sectional Survey and Quasi-Randomized Vignette Study

**DOI:** 10.2196/104151

**Published:** 2026-09-30

**Authors:** Julia-Astrid Moldt, Teresa Festl-Wietek, Kay Nieselt, Susanne Zabel, Manfred Claassen, Samuel Wagner, Ulrike Keim, Anne Herrmann-Werner

**Affiliations:** 1 TIME – Tübingen Institute for Medical Education Faculty of Medicine University of Tübingen Tübingen Germany; 2 Interfaculty Institute of Microbiology and Infection Medicine University of Tübingen Tübingen Germany; 3 Department of Computer Science University of Tübingen Tübingen Germany; 4 Department of Internal Medicine I University Hospital Tübingen Tübingen Germany; 5 M3 Research Center University Hospital Tübingen Tübingen Germany; 6 Institute for Health Sciences Department of Midwifery Science University Hospital Tübingen Tübingen Germany; 7 Research Institute for Women´s Health Department of Midwifery Science University Hospital Tübingen Tübingen Germany; 8 German Center for Mental Health (DZPG) Partner Site Tübingen Tübingen Germany

**Keywords:** AI, clinical decision-making, professional identity, medical students, physicians, explainability, trust, responsibility

## Abstract

**Background:**

The integration of AI into health care is increasingly shaping clinical practice and decision-making. Beyond technical performance, AI has implications for professional roles, clinical reasoning, and responsibility. Understanding how medical students and clinicians perceive AI, how these perceptions relate to professional identity concerns, and how clinicians evaluate AI-supported decisions in different clinical contexts is therefore important for both implementation and medical education.

**Objective:**

This study aimed to examine (1) how medical students and clinicians perceive the impact of AI on medical practice and professional identity and (2) how clinicians evaluate explainability, trustworthiness, and responsibility in AI-supported clinical decision-making across different contexts.

**Methods:**

A 2-phase quantitative study was conducted. In phase 1, medical students (n=93) and clinicians (n=65; N=158) completed a cross-sectional online survey assessing expectations regarding AI, perceived importance of AI across medical domains, and perceived professional identity threat. In phase 2, a separate sample of clinicians (N=68) was quasi-randomly assigned to 1 of 3 clinical vignettes (Watson, Triage, and OncoGuide) and evaluated AI-supported decisions along the dimensions of explainability, trustworthiness, and responsibility.

**Results:**

In phase 1, participants reported generally positive expectations regarding factual-level impacts of AI on medical practice. However, these expectations were not significantly associated with perceived professional identity threat. Medical students reported significantly higher identity threat than clinicians, despite largely similar expectations regarding AI’s factual and social impact. Perceived importance of AI across medical domains was associated with more positive expectations toward AI-supported medical practice, but not with identity threat. In phase 2, clinicians’ evaluations of AI-supported decisions varied across contexts, with explainability ratings differing significantly across vignette scenarios (*P*=.007), with lower ratings in the Triage vignette than in the Watson and OncoGuide vignettes. Trustworthiness and responsibility did not differ significantly across scenarios.

**Conclusions:**

The findings suggest that positive expectations regarding AI may coexist with professional identity concerns, while identity threat differed between medical students and clinicians in the present sample. At the same time, explainability evaluations differed across clinical decision contexts, whereas trustworthiness and responsibility remained comparatively stable. Together, these findings point to both professional identity-related and contextual considerations in AI implementation that may not be captured by expectations regarding technological benefits alone. Considering these dimensions may be relevant for implementation strategies and medical education.

## Introduction

### Background

The integration of AI into health care is increasingly framed as a transformative development with profound implications for medical practice, professional expertise, and clinical decision-making [1]. Recent advances in machine learning, predictive modeling, and algorithm-based clinical decision support are progressively moving from research prototypes into routine clinical workflows, particularly in domains such as radiology, pathology, oncology, and risk stratification [2-4]. As a result, clinicians increasingly need to balance algorithmic outputs with clinical judgment and professional accountability [5]. This has shifted attention from technical performance alone to the practical conditions under which AI recommendations can be meaningfully interpreted, justified, and integrated into clinical decision-making [6,7]. Rather than referring only to full technical transparency, explainability is increasingly discussed as context-sensitive information that supports clinicians in assessing the relevance, risks, and justification of AI-supported recommendations [8]. This reflects a broader shift in the literature, which increasingly frames AI implementation in medicine not merely as a technological innovation, but as a sociotechnical process involving clinical, organizational, ethical, and legal dimensions [9-11]. Contemporary research further suggests that AI adoption may affect health care professionals’ sense of professional identity, trust in clinical decision support, and negotiations of roles and responsibilities in clinical contexts [12,13]. Research exploring the social dimensions of AI integration in health care further demonstrates that AI implementation involves navigating organizational, professional, and ethical challenges, not merely enhancing algorithmic performance [14,15]. Against this background, understanding how clinicians perceive, interpret, and negotiate AI-supported decisions in concrete clinical situations becomes crucial.

Drawing on social constructivist approaches to technology, AI systems can be understood as open to different interpretations by relevant stakeholder groups within medicine. Their perceived meaning and implications depend not only on technical performance, but also on how clinicians and future clinicians position these systems in relation to expertise, authority, and responsibility [16,17]. The introduction of AI systems capable of generating diagnostic or therapeutic recommendations may challenge these foundations by redistributing cognitive tasks and raising questions about what is regarded as a legitimate source of clinical knowledge or decision authority [18,19]. For many practitioners, AI-generated outputs introduce new forms of uncertainty and raise questions about who or what constitutes a legitimate source of clinical knowledge, with implications for professional identity, trust, and accountability in everyday practice [20,21]. Empirical research shows that both medical students and practicing clinicians acknowledge the growing relevance of AI for medical practice [22]. Comparing these groups is particularly relevant because prior research suggests that medical students and less experienced clinicians may experience AI-related professional identity threats differently from experienced clinicians [23]. At the same time, perceptions are markedly ambivalent. While some view AI as a supportive tool that may enhance efficiency and clinical reasoning, others express concerns regarding deskilling, loss of professional autonomy, and shifting responsibilities [24,25]. Previous studies suggest that these perceptions differ across professional subgroups, training stages, and clinical specialties [22,26]. Beyond general attitudes toward AI, recent literature highlights explainability, trustworthiness, and questions of accountability and responsibility as important considerations in the clinical use of AI systems [20,27]. These aspects are closely connected to whether clinicians can understand and critically assess AI-supported recommendations, regard them as sufficiently reliable, and retain responsibility for the resulting clinical decision. They therefore informed the 3 evaluative dimensions examined in phase 2: explainability, trustworthiness, and responsibility. Trusting an AI system, demanding explanations, and retaining responsibility for AI-supported decisions are therefore not only technical or ethical issues but also relate to how clinicians perceive their role, authority, and accountability in clinical practice [28,29]. Recent empirical studies also suggest that the design and integration of AI-based decision support systems can influence clinicians’ trust while simultaneously raising concerns about professional identity and responsibility [30]. Closely related work by Ackerhans et al [13] examined how process design features of a fictitious sepsis-related AI-based clinical decision support system, including explainability, workflow integration, and system-induced accountability, shaped trust and perceived professional identity threat in a controlled scenario-based experimental setting. This provides important evidence on how system design features matter for trust and identity-related concerns. This study addresses a complementary perspective by shifting attention to variation across clinical decision contexts and to broader professional expectations and identity-related concerns surrounding AI. In phase 1, medical students and clinicians were examined with regard to general AI-related expectations and professional identity concerns. Separately, phase 2 examined clinicians’ evaluations of explainability, trustworthiness, and responsibility across distinct clinical vignette contexts.

### Research Questions

We address the following research questions (RQs):

- RQ 1: How do medical students and clinicians perceive the role of AI in medicine, particularly regarding expected impacts on medical practice and perceived professional identity threat, and do these perceptions differ between the 2 groups?
- RQ 2: Do clinicians’ evaluations and prioritizations of explainability, trustworthiness, and responsibility in AI-supported clinical decisions differ across clinical vignette contexts?

### Hypotheses

To address these RQs, we formulated the following hypotheses, which were preregistered as described in the Methods section. Hypotheses 1-3 relate to RQ1 and focus on general perceptions of AI and professional identity, whereas hypothesis 4 is stated here in an analysis-aligned form addressing RQ2 through situational evaluations of AI-supported clinical decisions across vignette contexts. Its broader preregistered formulation is reported in the Data Processing and Statistical Analysis section.

- Hypothesis 1: Clinicians and medical students who hold more positive expectations about AI’s impact on medical practice (eg, improved diagnosis and streamlined workflows) will report lower levels of perceived identity threat (measured across distinctiveness, continuity, self-esteem, and self-efficacy).
- Hypothesis 2: Participants who consider AI to be important across core medical domains (eg, diagnosis, patient communication, and administration) will show higher acceptance of AI and lower identity threat.
- Hypothesis 3: Medical students and clinicians differ in how they perceive AI’s factual and social impact.
- Hypothesis 4: Clinicians’ evaluations of explainability, trustworthiness, and responsibility in AI-supported clinical decision-making differ across clinical vignette contexts.

## Methods

### Study Design

The overall study design is illustrated in Figure 1. The study followed a sequential, 2-phase design combining a cross-sectional online survey (phase 1) and a quasi-randomized vignette study (phase 2). Phase 1 assessed general orientations toward AI and its perceived implications for the professional role and identity of clinicians (addressing RQ1 and hypotheses 1-3). Phase 2 complemented phase 1 by addressing the additional RQ2 in a separate clinician sample, focusing on context-specific evaluations of explainability, trustworthiness, and responsibility in AI-supported clinical decision-making.

**Figure 1:**
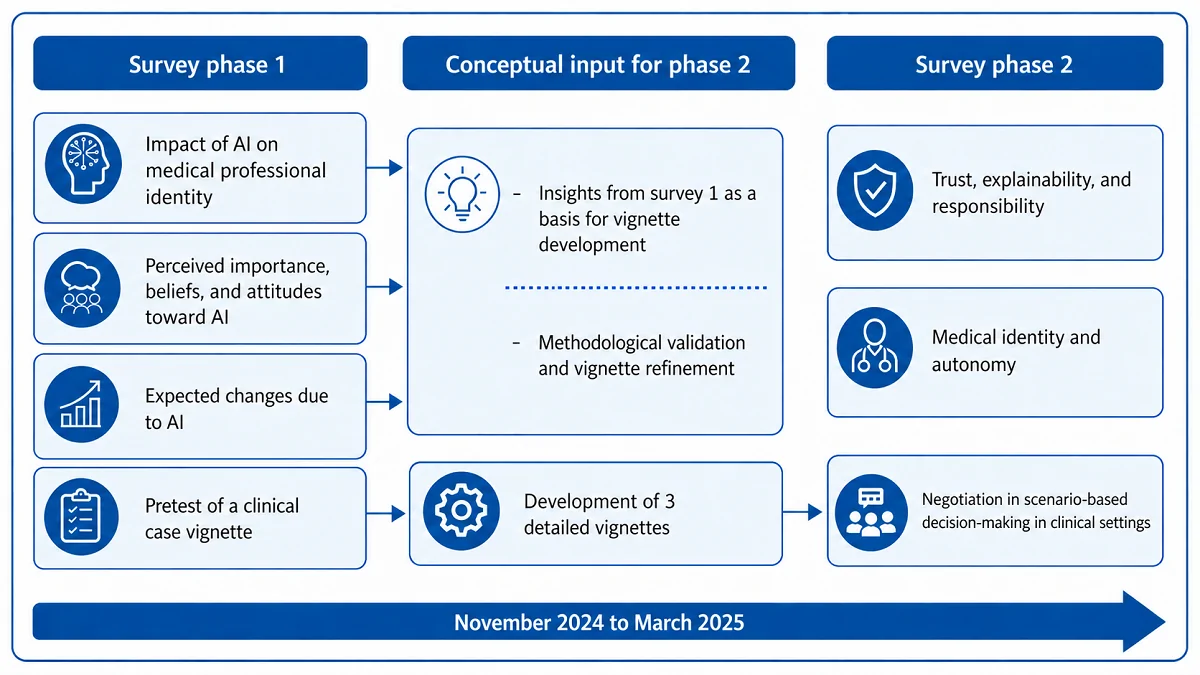
Survey outline.

### Survey Administration

Both phases were conducted as web-based surveys. Reporting of both survey phases was guided by the CHERRIES (Checklist for Reporting Results of Internet E-Surveys) [31]. The completed CHERRIES checklist is provided as Multimedia Appendix 1.

### Phase 1: Medical Students and Clinicians

Phase 1 was conducted between November 6, 2024, and February 9, 2025, using a study link [30]. It was an open web-based survey, as participation was possible for eligible individuals who received access to the study link through the recruitment channels described below. The questionnaire was developed based on a structured literature review and pretested with 13 participants from the target population. Minor revisions were made to improve clarity, comprehensibility, and technical functionality before fielding the final survey.

Recruitment was carried out through multiple channels relevant to the German medical education and health care context. The survey targeted medical students across different stages of training and clinicians with varying levels of professional experience and specialization. Central contact points at university hospitals, maximum-care hospitals, and medical student councils of human medicine were contacted directly and asked to disseminate the study invitation to potentially eligible participants. In addition, the study was distributed through internal institutional mailing lists and flyers. The invitation included information on the study purpose, target group, voluntary participation, approximate completion time, and a link to the web-based questionnaire. Participation was self-selected.

Eligible participants were medical students studying in Germany and clinicians working in Germany. Medical students included participants across all educational phases, including preclinical, clinical, and final-year training. The clinician group included practicing clinicians, clinical researchers, academics, and medically trained administrators.

To ensure consistency with the study’s focus on the German context of medicine and medical training, the dataset was cleaned based on predefined exclusion criteria. Participants who did not report studying or working in Germany were excluded. Only participants who completed the questionnaire up to the section immediately preceding the clinical case vignette were retained for the main analysis, as this indicated a sufficiently complete response set covering the core constructs of interest, including professional identity, perceptions of AI, and role-related expectations. Participants whose professional status could not be clearly assigned to either the medical student or clinician group were excluded from group-based analyses.

### Phase 2: Clinicians

Phase 2 was conducted as a web-based vignette survey between February 6 and March 20, 2025. The survey was accessed via a SoSci Survey study link and distributed to a defined group of clinicians participating in the medical didactics qualification program at the Tübingen Institute for Medical Education. The qualification program was held on several course dates during the data collection period, with different clinicians participating on different dates.

This phase used a purposive convenience sample of clinicians with teaching experience and an interest in medical education. Eligible participants were clinicians enrolled in the qualification program during the data collection period. The study invitation was distributed within the course context and included information on the study purpose, voluntary participation, data protection procedures, approximate completion time, and access to the web-based vignette survey.

The vignette-based survey was implemented as a course-related reflection task; however, participation in the research study was voluntary. After accessing the questionnaire, participants completed 1 assigned clinical vignette and a standardized set of follow-up items. The sample was self-selected, as participation depended on clinicians’ decision to complete the questionnaire.

### Measures

All measures were selected and developed to address the RQs and hypotheses of the study. Phase 1 measures focused on general perceptions of AI and its implications for professional identity (RQ1; hypotheses 1-3), whereas phase 2 measures captured situational evaluations of AI-supported decision-making in vignette-based scenarios (RQ2; hypothesis 4).

#### Initial Survey (Phase 1)

##### Questionnaire Development

The phase 1 questionnaire was developed based on a structured literature review and pretested with 13 participants from the target population. Minor revisions were made to improve clarity and conceptual coherence. The final instrument comprised closed-ended items assessing professional identity threat, perceived importance of AI across medical domains (IM), and expected AI-related changes in medical practice. The full set of phase 1 questionnaire items and response scales is provided in Multimedia Appendix 2.

##### Perceived AI Impact on Professional Identity

Perceived AI-related impacts on professional identity were assessed using 6 items theoretically informed by Breakwell’s identity process theory [32]: distinctiveness (1 item), continuity (1 item), self-esteem (2 items), and self-efficacy (2 items). The items focused on potentially threatening implications of AI for professional identity, such as changes in perceived expertise, confidence, and professional role. Items were rated on a 5-point agreement scale and averaged to form an overall perceived AI impact on professional identity (PI) score, with higher scores indicating stronger perceived professional identity threat. As the individual principles were represented by only 1 or 2 items, dimension-specific subscale scores were not constructed. Interitem correlations are reported in Multimedia Appendix 3. PI served as the primary outcome variable for hypothesis 1 and as one of the outcomes examined in hypothesis 2.

##### Perceived Importance of AI Across Medical Domains

IM was assessed across 5 medical domains: diagnostic accuracy and efficiency, support in complex diagnostic cases, personalized treatment planning, doctor-patient communication, and administrative simplification. Items were rated on a 3-point response scale (1=extremely to 3=not at all). For analysis, items were recoded so that higher values indicated higher IM and were averaged to obtain an overall index score of perceived importance across the selected domains. The index score was calculated from the available item responses when at least 1 of the 5 items had been completed; missing item responses were not imputed. Because these domains represent heterogeneous areas of medical practice, the measure was treated as a domain-level index rather than as a reflective scale measuring a single latent construct. The IM index was used in the analyses testing hypothesis 2.

##### Expected AI-Related Changes in Medical Practice

Expectations regarding AI-related changes in medical practice were assessed at 2 levels: factual-level expectations regarding AI-related changes in medical practice (EC_FL; 6 items), referring to clinical and organizational aspects of medical work, and social-level expectations regarding AI-related changes in medical practice (EC_SL; 9 items), referring to relational aspects of care. Items were rated on a 5-point agreement scale, with higher values indicating stronger agreement with the expected changes. Mean scores were calculated separately for EC_FL and EC_SL, with higher scores reflecting more positive expectations regarding AI-related changes at the respective level. One negatively worded social-level item (EC06_01) was reverse-coded so that higher values consistently reflected more positive expectations regarding AI-related social changes. Because phase 1 did not include a separate global acceptance measure, EC_FL was used in hypothesis 2 as a pragmatic acceptance-related indicator. In this context, the term refers to favorable evaluations of expected AI-related changes in practical aspects of medical work, rather than to direct behavioral acceptance. EC_FL was also used as the predictor in hypothesis 1, whereas both EC_FL and EC_SL were included in the group comparisons testing hypothesis 3.

##### Control Variables

Sociodemographic variables included role (medical student vs clinician), age, and professional experience. In addition, self-reported experience with AI in the medical field was assessed and included as a control variable in the analyses.

At the end of the phase 1 questionnaire, an exploratory clinical case vignette (“Watson”) was included to obtain initial insights into AI-supported clinical decision-making. Responses to this exploratory vignette were analyzed descriptively and used to inform the development of the quasi-randomized vignette study in phase 2.

#### Quasi-Randomized Vignette (Phase 2—RQ2; Hypothesis 4)

Phase 2 used a quasi-randomized vignette design to examine clinicians’ evaluations of AI-supported clinical decision-making in concrete situations. Participants were allocated to 1 of 3 clinical vignettes depicting distinct AI-supported decision scenarios using a quasi-random birth-date criterion based on their day of birth within the month: days 1-10 to Watson, days 11-20 to Triage, and days 21-31 to OncoGuide (Table 1). The vignette stimulus texts were kept comparable in structure and length. Excluding the subsequent rating items, vignette length ranged from approximately 238 to 317 words across the 3 conditions. Vignette-specific reading time was not recorded separately.

**Table 1:** Overview of vignettes and contextual dimensions.

Vignette	Clinical focus	Contextual variation	Key thematic dimension
Watson—intensive care	AI recommends a treatment that deviates from clinical guidelines	Limited transparency of algorithmic reasoning	Guideline adherence versus AI reasoning
Triage emergency	Team must make a rapid decision under time pressure	Diverging opinions between clinicians and AI	Trust under uncertainty and time pressure
OncoGuide—oncology	Shared decision-making between physician, patient, and AI	AI provides an individualized but nonguideline-based recommendation	Responsibility and patient autonomy

The vignettes were designed to capture typical situations of professional tension and negotiation that may arise with AI implementation (eg, algorithmic recommendations deviating from clinical guidelines, disagreement between clinicians and AI under time pressure, and AI involvement in shared decision-making with patients). After reading the assigned vignette, participants rated a standardized set of items assessing perceived trustworthiness, explainability, and responsibility of the AI system. All items were rated on 5-point Likert scales (1=strongly disagree to 5=strongly agree).

Some items referred to willingness to accept, follow, or implement an AI recommendation; however, these items were embedded within the dimension-specific scales or vignette-specific trade-off items and were therefore not treated as a separate acceptance outcome. Items belonging to each dimension were aggregated into 3 composite scale scores by calculating mean values. In addition to these standardized scales, vignette-specific prioritization items were included to capture how participants weighed these dimensions against each other within the concrete clinical context, for example, prioritizing trust over explainability under time pressure. These items reflected explicit trade-offs between dimensions and were analyzed descriptively: (1) Trust over explainability (eg, reliance on AI under time pressure), (2) responsibility over explainability (eg, clear accountability reduces need for transparency), and (3) explainability over trust (eg, refusal to follow AI advice without understanding). Although the wording was adapted to each vignette, the underlying dimension contrasts remained identical. All vignette scenarios and item wordings are provided in Multimedia Appendix 4.

### Data Processing and Statistical Analysis

The study was preregistered on the Open Science Framework, including the key hypotheses and planned analytic strategy [33]. The analyses for hypotheses 1 and 3 corresponded to the preregistered hypotheses, although the covariates included in the adjusted hypothesis 1 regression model were not specified in detail in the preregistration. For hypothesis 2, EC_FL were used as an acceptance-related indicator in phase 1. The preregistered formulation of hypothesis 4 stated that acceptance of AI-supported clinical decisions depends on perceived trustworthiness, explainability, and responsibility. In phase 2, acceptance of AI-supported clinical decisions was operationalized through participants’ agreement with dimension-specific items on explainability, trustworthiness, and responsibility, together with vignette-specific items referring to following, accepting, or implementing AI-supported recommendations. Higher ratings indicated stronger agreement with the respective acceptance-related evaluation in each vignette context. The phase 2 analyses examined whether ratings of explainability, trustworthiness, and responsibility differed across the Watson, Triage, and OncoGuide scenarios using one-way ANOVAs. No separate composite acceptance score was constructed; therefore, phase 2 is reported as a vignette-based analysis of acceptance-related evaluations rather than as a regression model predicting a global acceptance outcome. All statistical analyses were conducted using SPSS Statistics (version 31; IBM Corp). Sensitivity power analyses were conducted using G*Power (version 3.1.9.7) for the phase 2 one-way ANOVAs [34]. Data were screened for completeness and plausibility prior to analysis. Descriptive statistics were calculated for all study variables.

Hypotheses 1 and 2 were examined using correlational and multiple regression analyses. For hypothesis 1, perceived professional identity threat was regressed on EC_FL, with group membership, age, and prior AI experience included as covariates in the adjusted model. For hypothesis 2, IM was examined in relation to EC_FL and perceived professional identity threat. Group differences hypothesized in hypothesis 3 were tested using 2-tailed independent-samples *t* tests or nonparametric equivalents where appropriate. For phase 2, one-way ANOVAs were used to examine whether clinicians’ ratings of perceived explainability, trustworthiness, and responsibility differed across the 3 vignette scenarios. Vignette-specific prioritization items were analyzed descriptively. Statistical significance was set at *P*<.05. Effect sizes were reported where appropriate. Analyses not specified in the preregistration were labeled as exploratory. Missing data were not imputed. Descriptive item-level analyses were based on available valid responses per item. Regression analyses used listwise deletion. Bivariate correlations were calculated using available valid pairs. Analytic sample sizes are reported for the main analyses and in Multimedia Appendix 5.

### Ethical Considerations

The study, including both phase 1 and phase 2, received ethics approval from the ethics committee of the Medical Faculty of the University of Tübingen (342/2024BO2). All participants in both phases were informed about the study purpose, data protection procedures, the voluntary nature of participation, and their right to discontinue participation during the survey without providing reasons and without disadvantages. Informed consent was obtained prior to participation. Data were collected anonymously and used solely for the purposes of this study. No identifying information is reported in this paper. No monetary incentives or financial compensation were provided.

## Results

### Study Population and Descriptive Characteristics (Initial Survey)

A total of 323 participants started the phase 1 survey. Of these, 172 reached the predefined completion threshold, defined as completion of the questionnaire up to the section immediately preceding the clinical case vignette. The remaining 151 records did not meet this completion criterion. Of the 172 sufficiently complete responses, 14 were excluded because participants did not report studying or working in Germany. Therefore, the final phase 1 sample comprised 158 participants, including 93 (58.9%) medical students and 65 (41.1%) clinicians. Medical students represented different stages of medical education, including preclinical, clinical, and practical year training. The clinician group included practicing clinicians, resident and senior clinicians, clinical researchers, medical educators, and medically trained professionals working in administrative or managerial roles. Descriptive characteristics of the phase 1 study population are presented in Table 2. Age and sex statistics are based on valid responses only.

**Table 2:** Demographic and professional characteristics of participants (N=158).

Characteristic and category			Medical students, n=93 (58.9%)		Clinicians, n=65 (41.1%)
**Age (years)**					
	Mean (SD)		25.3 (5.4)		37.5 (9.7)
	Range		19-57		24-60
**Sex, n (%)^a^**					
	Female		46 (61.3)		24 (44.4)
	Male		29 (38.7)		30 (55.6)
**Experience**					
	Semester of study, mean (SD)		8.2 (3.8)		N/A^b^
	**Professional experience, n (%)^c^**		N/A		
		Less than 1 year		7 (14.0)	
		1-3 years		12 (24.0)	
		4-6 years		6 (12.0)	
		7-10 years		7 (14.0)	
		More than 10 years		18 (36.0)	

^a^Percentages are based on nonmissing responses; sex data were available for 75 medical students and 54 clinicians.

^b^N/A: not applicable.

^c^Percentages for professional experience are based on participants with available data (n=50).

### Phase 1: Initial Survey Results (RQ1; Hypotheses 1-3)

#### Descriptive Statistics and Measurement Properties

Table 3 presents descriptive statistics and internal consistency estimates for the phase 1 measures. Cronbach α values for the reflective multi-item scales ranged from 0.71 to 0.82. For the domain-level IM index, Cronbach α was 0.55 and is reported descriptively rather than as an indicator of scale homogeneity. Complete item-level descriptive statistics and interitem correlations for the PI and IM measures are provided in Multimedia Appendix 3.

**Table 3:** Descriptive statistics and internal consistency of main survey measures (phase 1).

Variable	For Cronbach α^a^, n	Items, n	Cronbach α	Mean (SD)	Range
Perceived AI impact on professional identity	141	6	0.71	3.13 (0.72)	1-5
AI importance across medical domains	135	5	0.55	2.38 (0.39)	1-3
Factual-level expectations regarding AI-related changes in medical practice	157	6	0.79	3.96 (0.64)	1-5
Social-level expectations regarding AI-related changes in medical practice	156	9	0.82	3.14 (0.65)	1-5

^a^Differences in ns reflect item-level nonresponse. ns for Cronbach α are based on complete cases using listwise deletion; item-level ns are reported in Multimedia Appendix 3.

To provide a more detailed view of the response patterns, selected item-level findings are reported below. Within the IM index, participants attributed the highest importance to the simplification of administrative tasks (mean 2.73, SD 0.48) and improvements in diagnostic accuracy and efficiency (mean 2.50, SD 0.50). This was followed by support in complex diagnostic cases (mean 2.39, SD 0.68) and the development of personalized treatment planning (mean 2.32, SD 0.70). In contrast, the perceived importance of AI for enhancing doctor-patient communication was comparatively lower (mean 1.93, SD 0.71). Participants reported varying levels of agreement regarding the potential impact of AI on their professional identity. The highest agreement was observed for the statement that AI will require continuous updates of medical skills to remain relevant (mean 3.93, SD 0.98). Moderate levels of agreement were found for concerns related to reduced confidence in clinical skills (mean 3.14, SD 1.30) and the possibility that AI errors could undermine clinical judgment (mean 3.05, SD 1.21). Lower levels of agreement were observed for items referring to a loss of uniqueness of medical expertise (mean 2.82, SD 1.13), weakened patient trust (mean 2.83, SD 1.19), and potential threats to professional reputation due to legal or ethical issues (mean 3.00, SD 1.17). Participants reported high agreement with EC_FL, particularly for real-time monitoring and early detection (mean 4.15, SD 0.87) and more efficient follow-up care (mean 4.08, SD 0.87). Expectations regarding diagnostic processes were also positive, as reflected in quicker diagnoses (mean 4.06, SD 0.82) and improved diagnostic accuracy (mean 3.90, SD 0.77). In contrast, expectations regarding social-level changes were more moderate. Lower agreement was observed for improvements in doctor-patient communication (mean 2.91, SD 1.13), while aspects such as patient understanding (mean 3.20, SD 1.16), clarity of medical information (mean 3.21, SD 1.04), and data exchange with colleagues (mean 3.62, SD 1.04) showed moderate agreement.

The following sections report the results of the hypothesis-driven analyses (hypotheses 1-3) examining associations between AI-related expectations, perceived importance, and PI, as well as group differences between medical students and clinicians.

#### Professional Identity Threat, AI-Related Perceptions, and Group Differences (RQ 1)

##### Hypothesis Overview

Hypothesis 1 focused on whether positive expectations about AI-related changes in medical practice were associated with perceived professional identity threat. Hypothesis 2 addressed a related but distinct question: whether perceiving AI as important across medical domains was associated with the acceptance indicator and identity threat. After examining the association-based hypotheses, we tested in hypothesis 3 whether medical students and clinicians differed in their EC_FL and EC_SL.

##### Hypothesis 1: Factual-Level AI Expectations and Perceived Identity Threat

To test hypothesis 1, we examined whether more positive EC_FL were associated with lower perceived professional identity threat. A hierarchical linear regression was conducted to examine predictors of perceived professional identity threat. In the final model, including factual-level AI expectations, group membership, age, and prior AI experience, the overall model was significant (adjusted *R*^2^=0.10; *F*_4,124_=4.59; *P*=.002; Table S1 in Multimedia Appendix 5). However, factual-level AI expectations were not a significant predictor of perceived identity threat (β=–.14; *P*=.11). Accordingly, hypothesis 1 was not supported. In the same model, group membership was significantly associated with perceived identity threat (β=–.31; *P*=.005), with clinicians reporting lower perceived identity threat than medical students. This was consistent with the unadjusted group comparison, in which medical students reported significantly higher perceived identity threat than clinicians (t_156_=4.02; *P*<.001; Cohen *d*=0.69). Age (β=–.06; *P*=.58) and prior AI experience (β=.07; *P*=.45) were not significantly associated with perceived identity threat. Collinearity diagnostics did not indicate problematic multicollinearity among the predictors (variance inflation factors=1.02-1.70; tolerance=0.59-0.99).

##### Hypothesis 2: Perceived Importance of AI Across Medical Domains

To test hypothesis 2, we examined whether IM was associated with 2 outcomes: an acceptance-related indicator, operationalized as EC_FL, and perceived professional identity threat. Pearson correlations indicated that IM was strongly associated with more positive factual-level AI expectations (*r*=0.68; *P*<.001), whereas no significant association was observed with perceived professional identity threat (*r*=–0.09; *P*=.29; Table S2 in Multimedia Appendix 5).

In a multiple linear regression model predicting the factual-level AI expectations, IM emerged as a strong and significant predictor (β=.71; *P*<.001), whereas group membership and prior AI experience were not significant predictors. In contrast, in a second regression model predicting perceived professional identity threat, IM was not a significant predictor (β=–.12; *P*=.17). Group membership remained a significant predictor, with clinicians reporting lower perceived identity threat than medical students. Thus, the expectation-related component of hypothesis 2 was supported, whereas the identity-threat component was not supported. Full regression results are provided in Table S3 in Multimedia Appendix 5.

##### Hypothesis 3: Group Differences in Factual-Level and Social-Level AI Expectations

To test hypothesis 3, we examined whether medical students and clinicians differ in their EC_FL and EC_SL. Two-tailed independent-samples *t* tests revealed no significant group differences for either dimension. For the factual-level expectations, medical students (mean 3.96, SD 0.62) and clinicians (mean 3.97, SD 0.66) did not differ (t_156_=–0.12; *P*=.91; Cohen *d*=–0.02). Likewise, no significant difference was observed for social-level expectations between medical students (mean 3.16, SD 0.63) and clinicians (mean 3.12, SD 0.68; t_155_=0.43; *P*=.67; Cohen *d*=0.07). Accordingly, hypothesis 3 was not supported.

### Phase 2: Quasi-Randomized Vignette Study (RQ2; Hypothesis 4)

#### Participant Allocation and Group Characteristics

The vignette study examined clinicians’ evaluations of AI-supported clinical decision-making across 3 distinct scenarios (Watson, Triage, and OncoGuide). A total of 68 clinicians participated and were quasi-randomly assigned to 1 of the 3 scenarios: Watson (n=24), Triage (n=21), and OncoGuide (n=23). The 3 vignette groups did not differ significantly in clinical specialty or prior experience with AI in medicine (Multimedia Appendix 6).

#### Vignette Analyses Related to Hypothesis 4: Explainability, Trustworthiness, and Responsibility Across Vignette Contexts

Phase 2 examined whether clinicians’ evaluations of explainability, trustworthiness, and responsibility differed across the 3 vignette scenarios. These analyses focused on the 3 evaluative dimensions specified in hypothesis 4. As shown in Table 4, explainability and responsibility were rated highly across scenarios, whereas trustworthiness received comparatively lower ratings. A one-way ANOVA showed a significant effect of vignette condition on perceived explainability (*F*_2,65_=5.41; *P*=.007; η^2^=0.14). Bonferroni-corrected post hoc tests indicated that explainability was rated lower in the Triage vignette (mean 3.92, SD 0.87) than in the Watson vignette (mean 4.47, SD 0.64; *P*=.03) and the OncoGuide vignette (mean 4.54, SD 0.50; *P*=.01). No difference was observed between the Watson and OncoGuide vignettes (*P*>.99). No significant differences between vignette conditions were observed for trustworthiness (*F*_2,65_=0.39; *P*=.68; η^2^=0.01) or responsibility (*F*_2,65_=1.01; *P*=.37; η^2^=0.03). A sensitivity power analysis indicated that, assuming α=.05 and 80% power, the phase 2 design with n=68 across 3 vignette groups could detect effects of approximately Cohen *f*=0.39, corresponding to η^2^≈0.13 using η^2^=*f*^2^/(1+*f*^2^). Thus, the analyses were primarily powered to detect comparatively large context effects, and smaller effects may have remained undetected. Taken together, only perceived explainability differed significantly across vignette contexts in the present sample.

**Table 4:** Mean (SD) for perceived explainability, trustworthiness, and responsibility across vignettes.

Dimension	Watson (n=24), mean (SD)	Triage (n=21), mean (SD)	OncoGuide (n=23), mean (SD)	Total (n=68), mean (SD)	Cronbach α^a^	*F* test (*df*=2, 65)	*P* value	η^2^
Explainability	4.47 (0.64)	3.92 (0.87)	4.54 (0.50)	4.32 (0.72)	0.59	5.41	**.**007	0.14
Trustworthiness	3.11 (0.98)	3.21 (0.76)	3.33 (0.82)	3.22 (0.86)	0.48	0.39	.68	0.01
Responsibility	4.36 (0.75)	4.16 (0.68)	4.45 (0.63)	4.33 (0.69)	0.35	1.01	.37	0.03

^a^Cronbach α by vignette (Watson/Triage/OncoGuide): explainability: 0.54/0.69/0.12; trustworthiness: 0.50/0.61/0.42; and responsibility: 0.36/0.39/0.26.

#### Prioritization Patterns Across Vignette Scenarios

Descriptive analyses examined how clinicians prioritized explainability, trustworthiness, and responsibility when these dimensions were in tension. Although the wording of the prioritization items was adapted to the respective scenario, the underlying dimension contrasts were identical across vignettes: agreement was highest for prioritizing explainability over trust, followed by prioritizing responsibility over explainability, while prioritizing trust over explainability received the lowest agreement (Table 5).

**Table 5:** Mean (SD) for prioritization trade-off items by vignettea.

Prioritization trade-off	Watson (n=24), mean (SD)	Triage (n=21), mean (SD)	OncoGuide (n=23), mean (SD)
Trust>explainability (time pressure/patient trust)	2.42 (1.18)	2.48 (1.18)	2.65 (1.27)
Responsibility>explainability (clear accountability)	3.33 (1.17)	3.10 (1.22)	3.52 (1.24)
Explainability>trust (transparency preference)	4.00 (1.18)	3.57 (1.03)	3.73 (1.42)

^a^Values represent mean agreement scores on a 5-point Likert scale (1=strongly disagree and 5=strongly agree). Items capture trade-offs between the perceived importance of trustworthiness, explainability, and responsibility in different clinical contexts.

Finally, exploratory analyses examined associations between individual dimension items and vignette-specific contextual items within each scenario using Spearman rank correlations. Only statistically significant correlations are reported in Multimedia Appendix 6. These analyses were exploratory and not part of the hypothesis-driven testing.

## Discussion

### Summary of Key Findings and Methodological Considerations

The study revealed a mix of significant and nonsignificant findings in both phases. In phase 1, participants reported positive EC_FL, while perceived professional identity threat remained moderate. Factual-level AI expectations were not significantly associated with perceived identity threat. IM was strongly associated with factual-level AI expectations, which served as an acceptance-related indicator, but it was not associated with perceived identity threat. Thus, the expectation-related component of hypothesis 2 was supported, whereas the identity-threat component was not supported.

This hypothesis 2 finding should be interpreted with some caution. The IM index covered different medical application areas rather than a narrowly defined construct, which is reflected in its low internal consistency. The strong association between this index and factual-level AI expectations may therefore partly reflect overlap between the measures, as both capture positive evaluations of AI’s role in practice. At the same time, the absence of an association with identity threat should not be taken as clear evidence that perceived importance and identity-related concerns are unrelated, as correlations may be weaker when reliability is low.

Hypothesis 3 was not supported, as medical students and clinicians did not differ significantly in EC_FL or EC_SL. The observed group difference in perceived identity threat should therefore be considered separately, as it emerged from the regression models predicting identity threat rather than from the hypothesis 3 test itself. In these models, group membership was significantly associated with identity threat, whereas factual-level expectations were not. In phase 2, clinicians rated explainability and responsibility highly across vignette scenarios, while trustworthiness received somewhat lower ratings. Explainability differed significantly between vignette contexts and was rated lower in the Triage vignette, which involved time pressure alongside other emergency decision-making features. Trustworthiness and responsibility did not differ significantly across scenarios. Because phase 2 did not include a separate acceptance outcome, these findings should not be interpreted as direct support for the broader preregistered acceptance-based formulation of hypothesis 4.

Before drawing theoretical conclusions, several methodological aspects should be taken into account. The null findings regarding identity threat may partly reflect measurement and design limitations. In addition, the phase 1 regression model explained only a modest share of variance in perceived identity threat, and the cross-sectional design does not allow conclusions about causal direction. For phase 2, the small vignette-specific subsamples limited statistical power to detect smaller context effects, particularly for trustworthiness and responsibility. The phase 2 composites comprised 3 items each that captured related but distinct aspects of explainability, trustworthiness, and responsibility. The comparatively low internal consistency estimates should therefore be interpreted in light of both the small number of items and the conceptual breadth of the dimensions. The phase 2 sample was also drawn from a single medical didactic qualification program, which may limit transferability to broader clinician populations. Therefore, the nonsignificant findings should not be interpreted as evidence of no association or no context effect, but rather as indicating that these associations and differences were not observed in the present sample and with the present measures.

### Divergence Between Task-Related Expectations and Identity-Related Concerns

Building on the methodological considerations outlined earlier, one plausible interpretation of the phase 1 findings is that task-related expectations and identity-related concerns refer to different evaluative layers of AI in medicine. The findings do not show that these dimensions are generally independent. Rather, they indicate that, in the present sample, positive factual-level expectations and IM were not associated with lower professional identity threat. A social constructivist perspective helps interpret this pattern without treating AI as a technology with fixed implications. From this perspective, the meaning of AI is negotiated within professional contexts and in relation to established understandings of expertise, decision-making authority, and responsibility. Building on insights from the social construction of technology, this interpretation focuses not only on evaluations of AI’s potential benefits but also on how these evaluations intersect with professional identity and role boundaries [12].

In phase 1, participants reported positive expectations regarding AI-related changes in medical practice, while perceived identity threat remained moderate and was not significantly associated with these expectations. Hypothesis 1 was therefore not supported. Similarly, perceiving AI as important across medical domains was strongly associated with more positive factual-level expectations (hypothesis 2), but it was not associated with lower identity threat. Thus, the expectation-related component of hypothesis 2 was supported, whereas the identity-threat component was not. This asymmetry suggests that perceiving AI as useful or important for clinical tasks does not necessarily resolve questions of professional role, judgment, and legitimacy. From a social-constructivist perspective, this pattern is plausible because AI is not interpreted only through its expected technical performance, but through the professional meanings attached to its use [35]. Participants could expect AI to improve diagnosis, monitoring, or workflow while still being concerned about how AI may affect clinical judgment, responsibility, or the perceived legitimacy of medical expertise. This distinction is relevant in light of prior research on AI adoption, which often highlights perceived usefulness as a key driver of acceptance [36]. Our findings support this view only for the expectation-related component: IM was associated with more positive factual-level expectations, but not with lower identity-related concerns. This is consistent with studies showing that health care professionals’ acceptance of AI depends not only on perceived usefulness but also on whether AI can be integrated into existing clinical practice and responsibility structures [37]. In this sense, identity-related concerns appear to reflect tensions around core elements of medical professionalism, including competence and clinical judgment rather than AI’s perceived usefulness alone [38]. This interpretation should be read in light of the methodological considerations outlined earlier. The findings do not provide confirmatory evidence for a stable decoupling between task-related expectations and identity-related concerns. Rather, they suggest that, in this sample, positive expectations toward AI did not necessarily coincide with lower identity threat.

Within this cautious interpretation, the item-level pattern helps specify what these identity-related concerns referred to. Participants reported high agreement with the need to continuously update medical skills, alongside concerns about reduced confidence in clinical skills and the potential for AI errors to undermine clinical judgment. These findings suggest that identity-related concerns may be tied to the conditions under which expertise is enacted and evaluated, rather than to attitudes toward AI per se. This is in line with prior work, showing that design and integration features of AI systems, including explainability, workflow integration, and accountability structures, can shape trust and identity-related concerns in partly nonparallel ways [13]. It also corresponds to research conceptualizing identity threat as a mechanism influencing technology use rather than a by-product of perceived usefulness [39]. Finally, the coexistence of strong expectations and persistent concerns resonates with prior stakeholder research, showing that AI in health care is framed both as a promise and as a source of uncertainty and ethical concern, reflecting implementation as a process of negotiated meaning rather than linear adoption [40]. Future studies should test this interpretation more directly using larger and more diverse samples, longitudinal or experimental designs, and refined measures of perceived AI importance across medical domains. Such designs would help distinguish whether the observed pattern reflects a divergence between task-related expectations and identity-related concerns, limited statistical power, measurement limitations, or sample-specific response patterns.

### Group Differences in Perceived Identity Threat

Hypothesis 3 was not supported, as medical students and clinicians did not differ significantly in EC_FL or EC_SL. Medical students and clinicians reported similarly positive EC_FL, particularly in relation to diagnostic processes and monitoring. However, medical students reported higher overall professional identity threat than clinicians, indicating greater concern about how AI may affect their developing sense of medical expertise and professional role. Thus, the observed group difference in identity threat was not accompanied by more negative expectations regarding AI’s practical contributions among medical students. Group membership remained significantly associated with identity threat after adjustment for age and prior AI experience. However, medical students and clinicians differed substantially in age and professional experience, and the present design does not allow these group-related characteristics to be clearly disentangled. Professional socialization therefore represents one possible interpretation of the observed group difference rather than an established explanatory mechanism. Professional identity in medicine is grounded in socially recognized expectations regarding expertise, decision authority, and responsibility. These expectations are acquired through professional socialization and stabilized in clinical practice, making questions of legitimate judgment and accountability central to the physician role [41]. From this perspective, AI does not simply introduce a new tool but may become relevant insofar as it interacts with these central aspects of professional practice. Professional identity formation (PIF) offers a useful perspective for interpreting the observed group difference. PIF conceptualizes medical training as a socialization process through which learners come to “think, act, and feel like a physician” [42,43]. In earlier stages of this process, expectations regarding legitimate expertise, authority, and professional responsibility are still being formed and negotiated. This interpretation is consistent with research on PIF, which emphasizes that during early stages of training, learners are particularly attentive to signals that define competence, authority, and legitimate participation within a profession [44,45]. Medical students’ greater concerns regarding their professional identity in relation to AI may therefore reflect heightened sensitivity to its implications for their evolving professional role, rather than generally more negative expectations toward AI. Previous research likewise suggests that positive expectations of medical AI may coexist with concerns about professional roles and responsibilities [27], while AI-supported decision-making may challenge established forms of clinical expertise, epistemic authority, and accountability [28]. Clinicians, in contrast, may interpret AI from within more established clinical role and responsibility structures [46]. However, the present study cannot determine whether the observed group difference is related to professional socialization, age, professional experience, or other differences between the groups. Prior work further suggests that sociotechnical arrangements can shape identity-related responses without necessarily being mirrored in other evaluations of AI [13]. AI use may also have implications for professional reputation. Recent findings suggest that individuals who use AI at work can be perceived as less competent or motivated, illustrating how the use of AI can become relevant not only for task performance but also for how professional competence is socially evaluated [47].

### Contextual Construction of Trust, Explainability, and Responsibility

Phase 2 examined clinicians’ evaluations of explainability, trustworthiness, and responsibility across 3 AI-supported clinical decision contexts, corresponding to the analysis-aligned formulation of hypothesis 4. The broader preregistered acceptance-based formulation and its operationalization are described in the Data Processing and Statistical Analysis section. The clearest difference was observed for explainability. Explainability was rated lower in the Triage vignette than in the Watson and OncoGuide vignettes. The Triage vignette described an emergency decision-making situation involving time pressure, diagnostic uncertainty, team-based assessment, and the need for rapid prioritization. In this context, full transparency of the AI’s reasoning may have appeared somewhat less central than in the other 2 scenarios. However, explainability was still rated highly overall, including in the Triage vignette. The lower explainability rating in the Triage vignette can be understood in relation to the different clinical contexts in which the same standardized explainability items were embedded. Across all scenarios, these items assessed whether clinicians considered it important to fully understand the basis of the AI’s decision, whether a comprehensible decision process increased their confidence in implementing the AI recommendation, and whether lack of transparency made accepting the recommendation more difficult. In the Watson and OncoGuide vignettes, the AI recommendation deviated from existing clinical guidelines. These scenarios also more explicitly involved questions of justification, responsibility, and, in the OncoGuide vignette, patient-facing shared decision-making. Explainability may therefore have been more closely linked to justification and defensibility in these 2 scenarios than in the Triage vignette. At the same time, the vignettes differed in several features simultaneously. The observed difference can therefore not be attributed to time pressure alone, but should be interpreted as a difference between vignette contexts. This fits with prior work, arguing that explanations are most relevant when they support clinical reasoning and accountable decision-making in context, rather than exhaustive transparency [6,48]. Responsibility was rated highly across all vignettes and did not differ significantly between scenarios. This may suggest that clinicians continued to locate accountability within the physician role even when AI systems were involved. However, phase 2 participants were recruited from a medical didactics qualification program and may therefore have been particularly attentive to questions of responsibility and reflective practice [49]. This may have contributed to the uniformly high responsibility ratings across the 3 vignette scenarios. These findings should therefore not be taken as evidence that responsibility is generally independent of clinical context. Smaller effects may have remained undetected. Nevertheless, the strong emphasis on physician responsibility is consistent with current governance and regulatory debates in health AI, which emphasize effective human oversight [50]. Trustworthiness was rated comparatively lower and showed less variation across scenarios. The exploratory correlation analyses suggest that trust may depend not only on general attitudes toward AI but also on the specific clinical situation and professional factors. This is consistent with review-based work, showing that trust in AI-based clinical decision support is shaped by factors such as system validation, workflow integration, transparency, and user experience [30]. Given the exploratory nature of these analyses and the small vignette-specific subsamples, these findings should also be interpreted cautiously. While these issues are often discussed as technical challenges, such as balancing model performance, explainability, and robustness [20,51], the vignette findings suggest a context-specific perspective. This raises the question of under which clinical conditions, explainability, trustworthiness, and responsibility become particularly relevant for clinicians’ acceptance of AI-supported recommendations.

### Implications for AI Implementation and Medical Education

Overall, the findings suggest that integrating AI into clinical practice calls for attention not only to performance but also to the role-related questions that AI raises around how decisions are justified and who remains accountable. In phase 1, participants reported positive expectations regarding AI’s practical contributions, while professional identity concerns remained present. This suggests that implementation efforts may benefit from addressing how responsibility, decision-making, and accountability are conceptualized in AI-supported care, rather than relying on benefit-oriented narratives alone [52]. For medical education, students reported higher perceived identity threat than clinicians despite similarly positive expectations. One possible interpretation is that, during professional socialization, AI may be encountered not only as a tool but also in relation to emerging understandings of valued expertise and legitimate decision authority in the physician role. These findings suggest that AI literacy may need to be complemented by educational approaches that address how AI-supported decisions are justified and how responsibility is assigned in clinical practice [43]. Phase 2 further indicates that explainability may need to be considered in a context-sensitive manner. Explainability differed across scenarios, suggesting that what counts as a sufficient explanation may depend on whether decisions must be justified against guidelines, made under time pressure, or discussed in patient-facing contexts. Taken together, these findings may inform context-sensitive approaches to oversight and justification in AI-supported decision-making, consistent with sociotechnical implementation perspectives [9].

### Limitations

Phase 1 was cross-sectional and therefore cannot show how expectations toward AI and perceived professional identity threat develop over time or influence each other causally. In addition, the index of perceived importance of AI across medical domains covered heterogeneous areas of medical practice and showed low internal consistency. Findings involving this index should therefore be read as referring to perceived importance across selected domains rather than to a single homogeneous construct. The 2 phases were conceptually linked but empirically distinct. Phase 1 examined general AI-related expectations and identity-related concerns in a sample of medical students and clinicians, whereas phase 2 examined vignette-based evaluations of explainability, trustworthiness, and responsibility in a separate sample. Phase 2 should therefore be understood as a complementary extension addressing RQ2, not as a direct empirical follow-up of phase 1.

Several limitations also concern the vignette study. The vignette-specific subsamples were small, which limited the ability to detect smaller differences between scenarios, particularly for trustworthiness and responsibility. In addition, participants were recruited from a single medical didactic qualification program. This recruitment context may have selected participants with a particular interest in teaching, reflection, and professional responsibility, which may have contributed to the high responsibility ratings across vignettes and limits transferability to broader clinician populations. Finally, phase 2 did not include a comparable medical student sample, so the findings cannot show how students would evaluate explainability, trustworthiness, and responsibility in similar vignette contexts.

No additional composite acceptance score was constructed in phase 2. The vignette findings therefore describe context-specific acceptance-related evaluations of explainability, trustworthiness, and responsibility, but should not be interpreted as a regression-based test of a global acceptance outcome. Because the study was conducted in Germany, the findings may also reflect specific institutional, educational, and medico-legal conditions and may not be directly transferable to other health care systems.

### Conclusions

This study suggests that positive expectations about AI’s task-related benefits do not necessarily coincide with lower professional identity concerns. In phase 1, both medical students and clinicians expressed largely positive EC_FL, and these expectations did not differ between groups. IM was associated with more positive factual-level AI expectations, but not with lower perceived professional identity threat. In addition, medical students reported higher perceived professional identity threat than clinicians.

In phase 2, which focused on clinicians, only perceived explainability differed significantly across vignette contexts. Explainability was rated lower in the Triage vignette than in the Watson and OncoGuide vignettes. Trustworthiness and responsibility did not differ significantly across vignettes; however, given the small vignette-specific subsamples, these nonsignificant findings should not be interpreted as evidence that these dimensions are context-invariant. Taken together, the findings suggest that the evaluation of AI-supported decision-making cannot be inferred from expected performance benefits alone. They may also depend on how AI is positioned in relation to clinical judgment, responsibility, and the justification of decisions in specific clinical contexts. This supports the view that AI implementation in health care is not only a technical process, but also raises questions about clinical expertise, decision-making, and responsibility.

## Data Availability

The data that support the findings of this study are available from the corresponding author (JAM) upon reasonable request.

## Appendix

Multimedia Appendix 1 CHERRIES checklist.

Multimedia Appendix 2 Initial survey (full item list).

Multimedia Appendix 3 Item-level descriptive statistics and interitem correlations.

Multimedia Appendix 4 Vignette scenarios and item wordings (phase 2: vignette study).

Multimedia Appendix 5 Supplementary statistical analyses for phase 1.

Multimedia Appendix 6 Additional phase 2 analyses and vignette group characteristics.

## Abbreviations

CHERRIES: Checklist for Reporting Results of Internet E-Surveys
EC_FL: factual-level expectations regarding AI-related changes in medical practice
EC_SL: social-level expectations regarding AI-related changes in medical practice
IM: perceived importance of AI across medical domains
PI: perceived AI impact on professional identity
PIF: professional identity formation
RQ: research question
