# Supplementary material for: AI Perceptions, Professional Identity, and AI-Supported Clinical Decisions Among Medical Students and Clinicians: Cross-Sectional Survey and Quasi-Randomized Vignette Study
Source: JMIR Med Educ. 2026 Sep 30;12:e104151. doi: 10.2196/104151 (PMC13669706; doi:10.2196/104151)
Supplement: Supplementary file 2 [file mededu_v12i1e104151_app2.docx]

# Initial Survey (full item list)

This appendix provides the full list of survey items used in the initial study (phase , grouped by construct.

**Current Employment/Education Status (SD03)**

Which of the following roles describe or apply to you most?

**SD03 Current Employment/Education Status**

1 = Medical Student (Preclinical/Vorklinik)
2 = Medical Student (Clinical/Klinik)
8 = Medical intern (Practical year/PJ)
3 = Attending Physician/Specialist (Facharzt/Ärztin)
4 = Researcher in a Clinical Setting
5 = Assistant/Resident physician (Assistenzarzt/Ärztin)
9 = Medical administrator/Health Manager (Leitender Arzt/Ärztin)
11 = Academic (Medical educator, professor, lecturer, ...)
7 = Other:
12 = None of the above
-9 = Not answered

**Perceived AI Impact on Professional Identity (PI)**

Please read the following statements about the potential impacts of Artificial Intelligence (AI) on your medical practice.

Indicate the extent to which you agree or disagree with each statement and how you believe AI might influence your professional role as a (future, if you are still a student) physician.

Response scale:
1 = completely disagree
2 = rather disagree
3 = neither agree nor disagree
4 = rather agree
5 = completely agree
-1 = not applicable
-9 = Not answered

**PI01_01** AI will make my skills less unique because it has the potential to take over many tasks that I perform.
**PI01_02** The implementation of AI in healthcare will require continuous skill updates to keep my medical expertise relevant.
**PI01_03** AI might weaken the trust and connection I’ve built with my patients by altering traditional patient-care dynamics.
**PI01_04** Legal and ethical issues with AI could affect my reputation and trustworthiness as a healthcare provider.
**PI01_05** Increased reliance on AI may reduce my confidence in my own clinical skills and decision-making abilities.
**PI01_06** The potential for AI to make incorrect diagnoses could undermine my ability to trust my clinical judgment.

**Perceived Importance of AI across medical domains (IM)**

In this section we are interested in understanding how you perceive the importance of AI in different medical domains.

Please indicate how much you believe AI will improve the following tasks:

Response scale:
1 = extremely
2 = moderately
3 = not at all
-1 = unsure
-9 = Not answered

IM01_01 The integration of AI in diagnostic processes will significantly enhance accuracy and efficiency.
IM01_02 AI will help in diagnosing complex cases that are challenging for doctors.
IM01_03 The integration of AI will be essential for developing personalized treatment plans.
IM01_04 AI will enhance communication between doctors and patients by providing real-time, data-driven insights.
IM01_05 AI is important for simplifying administrative tasks, allowing more time for patient care.

* For scale construction, AI importance items were recoded so that higher values indicate higher perceived importance.

**Factual-Level Expectations Regarding AI-Related Changes in Medical Practice (EC_FL)**

"In the following, we build on the idea that the increasing integration of AI into healthcare is

reshaping the role of doctors by supporting or even taking over tasks such as diagnosis and

treatment planning or affecting the doctor-patient-relationship."

Please reflect on how you believe AI might change medical practice in the near future.

Response scale:
1 = completely disagree
2 = rather disagree
3 = neither agree nor disagree
4 = rather agree
5 = completely agree
-9 = Not answered

EC01_01 AI systems will significantly improve the accuracy and speed of medical diagnoses.
EC01_02 AI-based diagnostic tools can lead to quicker diagnoses compared to traditional methods.
EC01_03 AI will significantly improve the development of personalized treatment plans for patients.
EC01_04 AI will provide valuable support in selecting effective therapies and making decisions in complex cases.
EC01_05 AI systems will enable real-time monitoring and earlier detection of changes in patients' health conditions.
EC01_06 AI will make follow-up care more efficient through advanced monitoring tools.

**Social-Level Expectations Regarding AI-Related Changes in Medical Practice (EC_SL)**

Response scale:
1 = completely disagree
2 = rather disagree
3 = neither agree nor disagree
4 = rather agree
5 = completely agree
-9 = Not answered

EC04_01 AI technologies will enhance communication between doctors and patients.
EC04_02 The use of AI in medicine will make it easier for patients to understand their medical conditions.
EC04_03 AI will allow doctors to spend more time on personal interactions with patients.
EC04_04 AI will improve the clarity and accuracy of the medical information communicated to patients.
EC04_05 AI will simplify the exchange of medical data with colleagues.
EC04_06 AI will lead to more standardized communication protocols in patient care.

EC06_01 I fear that the use of AI could reduce empathy in doctor-patient communication.
EC06_02 I believe that AI will build trust between patients and the healthcare system by providing reliable information.
EC06_03 I expect AI to improve communication and collaboration within the medical team.

**SD30 Control question**

"How much experience have you already gained with AI in the medical field?"

SD30 Control question
1 = No experience (e.g., no practical experience using AI in medicine, but may have heard about it.)
2 = Little experience (e.g., attended a lecture or training on AI in medicine)
3 = Some experience (e.g., occasional use of AI in medical practice or attended multiple trainings)
4 = Extensive experience (e.g., regular use of AI in medical practice or involved in research/development in this area)
-9 = Not answered

**Sociodemographic Variables (SD)**

SD01 Gender (zum kombinieren)
1 = Female
2 = Male
3 = Diverse
4 = Prefer not to disclose
-9 = Not answered

SD02_01 I am ... years old.

SD03 Current Employment/Education Status

1 = Medical Student (Preclinical/Vorklinik)
2 = Medical Student (Clinical/Klinik)
8 = Medical intern (Practical year/PJ)
3 = Attending Physician/Specialist (Facharzt/Ärztin)
4 = Researcher in a Clinical Setting
5 = Assistant/Resident physician (Assistenzarzt/Ärztin)
9 = Medical administrator/Health Manager (Leitender Arzt/Ärztin)
11 = Academic (Medical educator, professor, lecturer, ...)
7 = Other:
12 = None of the above
-9 = Not answered

SD04 Medical experise

1 = Medicine
2 = Nursing
3 = Midwifery
4 = Medical Technology
5 = Pharmacy
6 = Biomedical Research
7 = Public Health
8 = Allied Health Professions (e.g. physiotherapy)
9 = Studying in a related healthcare field
10 = Studying in a non-helathcare field
11 = Currently employed outside of healthcare
-9 = Not answered

**SD05 Study field / Semester**

"Which subject are you studying and in which semester are you in?"

SD05_01 I study ... and
SD05_02 I´m in my ... semester.

**SD07 Highest Educational Qualification**
1 = Abitur (or equivalent high school diploma)
13 = Bachelor’s degree (if applicable)
17 = Mater's degree (if applicable)
14 = Vocational Training
15 = State Examination (Staatsexamen in Medicine)
16 = Doctoral degree (e.g., Dr. med., PhD)
12 = Other degree:
-9 = Not answered

**SD08 Years of Professional Experience (for employed participants)**
1 = Less than 1 year
2 = 1–3 years
3 = 4–6 years
4 = 7–10 years
5 = More than 10 years
-9 = Not answered

**SD09 working field**

"What is your current employment situation?"

SD09_01 I am working since ... years
SD09_02 as ... .
SD09_03 The specific field of specialization is ... .

**SD11 working sector**

1 = Academic
2 = Corporate
3 = Government
4 = Healthcare
5 = Other
-9 = Not answered
