# Supplementary material for: AI Perceptions, Professional Identity, and AI-Supported Clinical Decisions Among Medical Students and Clinicians: Cross-Sectional Survey and Quasi-Randomized Vignette Study
Source: JMIR Med Educ. 2026 Sep 30;12:e104151. doi: 10.2196/104151 (PMC13669706; doi:10.2196/104151)
Supplement: Supplementary file 3 [file mededu_v12i1e104151_app3.docx]

Item-Level Descriptive Statistics and Inter-Item Correlations

Table S1. Item-level descriptive statistics for perceived importance of AI across medical domains (IM)

| **Item** | **Description** | **n** | **Mean (SD)** |
| --- | --- | --- | --- |
| IM01_01 | AI will enhance accuracy and efficiency in diagnostic processes | 152 | 2.50 (0.50) |
| IM01_02 | AI will help in diagnosing complex cases that are challenging for doctors | 152 | 2.39 (0.68) |
| IM01_03 | AI will be essential for developing personalized treatment plans | 154 | 2.32 (0.70) |
| IM01_04 | AI will enhance doctor–patient communication through real-time insights | 141 | 1.93 (0.71) |
| IM01_05 | AI will simplify administrative tasks, allowing more time for patient care | 154 | 2.73 (0.48) |

Table S2. Inter-item correlations for the perceived importance of AI across medical domains index (IM)

| **Item** | **Statistic** | **1** | **2** | **3** | **4** | **5** |
| --- | --- | --- | --- | --- | --- | --- |
| 1. Diagnostic accuracy and efficiency | *r* | — |  |  |  |  |
|  | *P* value | — |  |  |  |  |
| 2. Complex diagnostic cases | *r* | 0.36 | — |  |  |  |
|  | *P* value | <.001 | — |  |  |  |
| 3. Personalised treatment planning | *r* | 0.20 | 0.31 | — |  |  |
|  | *P* value | .019 | <.001 | — |  |  |
| 4. Doctor–patient communication | *r* | 0.18 | 0.11 | 0.34 | — |  |
|  | *P* value | .036 | .220 | <.001 | — |  |
| 5. Administrative tasks | *r* | 0.07 | −0.04 | 0.13 | 0.27 | — |
|  | *P* value | .434 | .667 | .135 | .001 | — |

^a^Values are Pearson correlation coefficients based on complete cases (N=135). IM items were recoded so that higher values indicate higher perceived importance of AI.

Table S3. Item-level descriptive statistics for perceived AI impact on professional identity (PI)

| **Item** | **Description** | **n** | **Mean (SD)** |
| --- | --- | --- | --- |
| PI01_01 | AI will make my skills less unique by taking over many of my tasks | 154 | 2.82 (1.13) |
| PI01_02 | AI in healthcare will require continuous skill updates to remain relevant | 152 | 3.93 (0.98) |
| PI01_03 | AI might weaken trust and connection with patients | 151 | 2.83 (1.19) |
| PI01_04 | Legal and ethical issues with AI may affect my professional reputation | 154 | 3.00 (1.17) |
| PI01_05 | Increased reliance on AI may reduce confidence in my clinical skills | 154 | 3.14 (1.30) |
| PI01_06 | AI errors could undermine my ability to trust my clinical judgment | 152 | 3.05 (1.21) |

Table S4. Inter-item correlations for the perceived AI impact on professional identity measure (PI)

| **Item** | **Statistic** | **1** | **2** | **3** | **4** | **5** | **6** |
| --- | --- | --- | --- | --- | --- | --- | --- |
| 1. Skills less unique (distinctiveness) | *r* | — |  |  |  |  |  |
|  | *P* value | — |  |  |  |  |  |
| 2. Continuous skill updates (continuity) | *r* | 0.31 | — |  |  |  |  |
|  | *P* value | <.001 | — |  |  |  |  |
| 3. Trust and connection with patients (self-esteem) | *r* | 0.31 | 0.10 | — |  |  |  |
|  | *P* value | <.001 | .234 | — |  |  |  |
| 4. Reputation and trustworthiness (self-esteem) | *r* | 0.20 | 0.24 | 0.52 | — |  |  |
|  | *P* value | .016 | .004 | <.001 | — |  |  |
| 5. Confidence in clinical skills and decision-making (self-efficacy) | *r* | 0.36 | 0.15 | 0.43 | 0.30 | — |  |
|  | *P* value | <.001 | .085 | <.001 | <.001 | — |  |
| 6. Trust in clinical judgement (self-efficacy) | *r* | 0.11 | 0.21 | 0.22 | 0.28 | 0.51 | — |
|  | *P* value | .189 | .012 | .008 | <.001 | <.001 | — |

**^a^** Values are Pearson correlation coefficients based on complete cases (N=141).

Table S5. Item-level descriptive statistics for Factual-Level Expectations Regarding AI-Related Changes in Medical Practice (EC_FL)

| **Item** | **Description** | **n** | **Mean (SD)** |
| --- | --- | --- | --- |
| EC01_01 | AI will significantly improve accuracy and speed of medical diagnoses | 157 | 3.90 (0.77) |
| EC01_02 | AI-based tools will lead to quicker diagnoses | 157 | 4.06 (0.82) |
| EC01_03 | AI will improve personalized treatment planning | 157 | 3.80 (1.03) |
| EC01_04 | AI will support therapy selection and complex decision-making | 157 | 3.85 (0.96) |
| EC01_05 | AI will enable real-time monitoring and earlier detection | 158 | 4.15 (0.87) |
| EC01_06 | AI will improve efficiency of follow-up care | 157 | 4.08 (0.87) |

Table S6. Item-level descriptive statistics for Social-Level Expectations Regarding AI-Related Changes in Medical Practice (EC_SL)

| **Item** | **Description** | **n** | **Mean (SD)** |
| --- | --- | --- | --- |
| EC04_01 | AI will enhance doctor–patient communication | 156 | 2.91 (1.13) |
| EC04_02 | AI will improve patient understanding | 157 | 3.20 (1.16) |
| EC04_03 | AI will allow more time for personal interaction | 156 | 3.74 (0.95) |
| EC04_04 | AI will improve clarity of communicated medical information | 156 | 3.21 (1.04) |
| EC04_05 | AI will simplify exchange of medical data with colleagues | 156 | 3.62 (1.04) |
| EC04_06 | AI will standardize communication protocols | 156 | 3.67 (0.99) |
| EC06_01 | I fear that the use of AI could reduce empathy in doctor-patient communication | 156 | 1.90 (0.70) |
| EC06_02 | I believe that AI will build trust between patients and the healthcare system by providing reliable information | 156 | 2.81 (0.99) |
| EC06_03 | I expect AI to improve communication and collaboration within the medical team | 157 | 3.20 (0.99) |
