# Supplementary material for: AI Perceptions, Professional Identity, and AI-Supported Clinical Decisions Among Medical Students and Clinicians: Cross-Sectional Survey and Quasi-Randomized Vignette Study
Source: JMIR Med Educ. 2026 Sep 30;12:e104151. doi: 10.2196/104151 (PMC13669706; doi:10.2196/104151)
Supplement: Supplementary file 4 [file mededu_v12i1e104151_app4.docx]

# Vignette Scenarios and Item Wordings (Phase 2: Vignette Study)

## Vignette Scenarios

**Vignette 1: Watson – AI-Supported Treatment Adjustment in Intensive Care**

Watson is an AI-based clinical decision support system that analyzes extensive patient data, including vital signs, laboratory values, and treatment histories. Based on these data, the system identifies patterns and generates treatment recommendations tailored to individual patients. The system was specifically developed to support treatment decisions for patients with sepsis, using data from intensive care units across multiple hospitals.

Using reinforcement learning, Watson has learned which treatment strategies are most effective in improving patient survival. Its recommendations are based on statistical success probabilities. In cases of incomplete data, the system uses statistical estimation methods to compensate for missing information. While Watson emphasizes its objectivity, the underlying decision-making process remains partly unclear.

You are an experienced physician working in the intensive care unit of a large hospital. You are currently treating a 58-year-old patient with severe sepsis of unclear origin.

The AI system recommends deviating from guideline-based antibiotic therapy and suggests an unconventional drug combination. This recommendation is based on analyses of thousands of similar cases in which this strategy improved survival rates.

However, your clinical experience suggests that this combination is rarely used in such cases, and existing guidelines recommend a different standard therapy. You are also unable to fully understand how the AI arrived at its recommendation.

While the system refers to comprehensive data analyses, it remains unclear which risks may be associated with deviating from established guidelines and what responsibility this entails for you. Although the AI system has been reliable in the past, you recall a similar case in which its recommendation was incorrect.

**Vignette 2: Triage Decision-Making in the Emergency Department**

In the emergency department of a large urban hospital, medical staff must make decisions under considerable time pressure. Initial patient assessment plays a crucial role in determining treatment priority.

An AI-based system is used to support triage decisions. It analyzes patient data such as vital signs, blood gas values, and prehospital reports and provides prioritized treatment recommendations along with a justification. The goal is to improve both speed and accuracy and to reduce errors due to stress or overload.

A 58-year-old female patient is admitted with non-specific abdominal pain, nausea, and general discomfort. Her vital signs are stable, but symptoms have gradually worsened.

The AI system classifies the patient as high priority and recommends immediate cardiac evaluation, citing an increased risk of atypical myocardial infarction in women. The recommendation is based on a large dataset of similar cases.

While your colleagues consider the AI recommendation convincing, you also consider alternative explanations, such as gastrointestinal causes. The emergency department is busy, and a quick decision is required. As the responsible physician, you must make the final decision while your team awaits your assessment.

**Vignette 3: Autonomy and Responsibility in Oncology**

The AI system “OncoGuide” is designed to generate personalized treatment recommendations for cancer patients. It uses large datasets including molecular profiles, treatment histories, and clinical studies to calculate individualized therapy options.

Recommendations are presented together with statistical probabilities of success and risk. However, the decision-making process remains difficult to fully understand due to the complexity of the algorithms and limited transparency of the data sources.

You are a senior oncologist treating a 55-year-old patient with metastatic breast cancer. After standard therapies have been exhausted, the system recommends an experimental immunotherapy.

The recommendation is based on molecular data and analyses of similar cases. While the system indicates a high probability of treatment response, it also highlights significant risks, including severe side effects.

The recommendation deviates from national guidelines and has only been tested in a limited number of cases. The patient asks for a clear recommendation and is willing to participate in the decision if risks and benefits are understandable.

You feel responsible for critically evaluating the recommendation while also bearing responsibility for the outcome.

## Standardized Evaluation Items (All Vignettes)

**Response scale:**
1 = strongly disagree
2 = rather disagree
3 = neither agree nor disagree
4 = rather agree
5 = completely agree
-9 = Not answered

**Explainability**

1. It is important for me to fully understand the basis of the AI’s decision before following its recommendation.
2. I feel more confident implementing the AI recommendation when I can fully understand its decision-making process.
3. A lack of transparency makes it difficult for me to accept the proposed treatment.

**Trustworthiness**

4. I trust the AI when its recommendations align with my previous experience.
5. The scientific basis and data underlying the AI strengthen my trust in its recommendations, even when they deviate from guidelines.
6. The trust of my colleagues in the AI influences my own willingness to follow its recommendations.

**Responsibility**

7. I take responsibility for the final decision, regardless of whether I follow the AI recommendation.
8. I feel obliged to critically evaluate the AI recommendation, even if it appears statistically advantageous.
9. I would only take responsibility for the consequences of an AI recommendation if it is transparently explained.

## Vignette-Specific Prioritization Items

**Response scale:**
1 = strongly disagree
2 = rather disagree
3 = neither agree nor disagree
4 = rather agree
5 = completely agree
-9 = Not answered

**Vignette 1: Watson (guideline deviation / transparency conflict)**

10. In time-critical situations, I rely more strongly on the recommendations of the AI, even if I do not fully understand the decision-making process.
11. I prioritize the recommendations of the AI more highly when the responsibility for possible consequences is clearly regulated.
12. Even if the AI recommendation is statistically successful, I would not follow it if I cannot understand its decision-making process.
13. I would only accept the recommendation of the AI if it is consistent with the guidelines.
14. The deviation of the AI from the guidelines makes me feel uncertain in my decision-making.
15. I have difficulty trusting the AI when its decision-making basis is not understandable to me.
16. I would be more likely to follow the AI if it explains its decision-making process in detail.
17. Guidelines represent the safest basis for medical decisions, regardless of the suggestions of the AI.
18. I prefer to rely on established guidelines rather than on AI-based recommendations.

**Vignette 2: Triage (time pressure / team / experience)**

10. In time-critical situations, I rely more strongly on the recommendations of the AI, even if I do not fully understand the decision-making process.
11. If the responsibility for the decision clearly lies with me and the need to act quickly is high, the explainability of the AI system’s suggestions is less relevant for me.
12. Even if the AI recommendation is statistically successful, I would not follow it if I cannot understand its decision-making process.
13. The trust of my colleagues in the AI influences my willingness to follow its recommendations.
14. I would be more likely to trust the AI if I see that my colleagues have had positive experiences with it.
15. My clinical experience gives me more certainty than the recommendations of the AI.
16. I trust my intuition more than the suggestions of the AI, especially in critical situations.
17. Under time pressure, I would rely more on the AI recommendations, even if I do not fully understand their decision-making process.
18. In stressful situations, the AI is a valuable support for making quick decisions.

**Vignette 3: OncoGuide (responsibility / patient involvement / autonomy)**

10. I prioritize the recommendations of the AI more highly when they are perceived by the patient as understandable and trustworthy.
11. I prioritize the recommendations of the AI more highly when the responsibility for possible consequences is clearly regulated.
12. Even if the AI recommendation is statistically successful, I would not follow it if I cannot understand its decision-making process.
13. The active involvement of the patient in decision-making influences how strongly I perceive AI as support.
14. If the patient can understand the AI recommendation, I am more willing to follow it.
15. If the AI recommendation deviates from the guidelines, I feel uncertain about how I should take responsibility.
16. The deviation of the AI from the guidelines calls into question my role as the primary decision-maker.
17. I see AI as a supportive tool, not as a replacement for my medical expertise.
18. The AI should only complement what human expertise cannot achieve and should not make decisions independently.
