# Supplementary material for: AI Perceptions, Professional Identity, and AI-Supported Clinical Decisions Among Medical Students and Clinicians: Cross-Sectional Survey and Quasi-Randomized Vignette Study
Source: JMIR Med Educ. 2026 Sep 30;12:e104151. doi: 10.2196/104151 (PMC13669706; doi:10.2196/104151)
Supplement: Supplementary file 5 [file mededu_v12i1e104151_app5.docx]

Phase 1 Regression and Correlation Analyses

***Table S1. Hierarchical linear regression predicting perceived professional identity threat (Phase 1)^a^***

| Predictor | B (SE) | β | *t* | *P* value |
| --- | --- | --- | --- | --- |
| (Constant) | 4.44 (0.45) | — | 9.87 | <.001 |
| Factual-level AI expectations | −0.16 (0.10) | −0.14 | −1.63 | .11 |
| Age (years) | −0.004 (0.008) | −0.06 | −0.56 | .58 |
| Group membership (clinicians vs medical students) | −0.44 (0.16) | −0.31 | −2.83 | .005 |
| Control question (prior AI experience) | 0.05 (0.07) | 0.07 | 0.76 | .45 |

^a^N=129. R²=0.13, adjusted R²=0.10, F(4,124)=4.59, P=.002. Dependent variable: perceived professional identity threat.

***Table S2. Pearson correlations between perceived importance of AI, factual-level AI expectations, and perceived professional identity threat^a^***

| Variable | 1 | 2 | 3 |
| --- | --- | --- | --- |
| 1. Perceived importance of AI across medical domains) | 1 | .68 (P<.001, N=157) | −0.09 (P=.29); N=157 |
| 2. Factual-level AI expectations | 0.68 (P<.001) | 1 | −0.08 (P=.29; N=158) |
| 3. Perceived professional identity threat | −0.09 (P=.29; N=157) | −0.08 (P=.30; N=158) | 1 |

^a^Values are Pearson correlation coefficients calculated using pairwise deletion.

**Table S3. Regression analyses examining associations between perceived importance of AI across medical domains, factual-level AI expectations (EC_FL; acceptance-related indicator), and perceived professional identity threat (H2)^a^**

| Predictor | Factual-level AI expectations |  | Perceived professional identity threat |  |
| --- | --- | --- | --- | --- |
|  | β | *P value* | β | *P value* |
| AI importance (medical domains) | .71 | < .001 | −.12 | .17 |
| Group membership (clinicians vs medical students) | −.04 | .53 | −.34 | < .001 |
| Prior AI experience (control question) | .08 | .23 | .05 | .53 |

| Model fit |  |  |  |  |
| --- | --- | --- | --- | --- |
| Adjusted R² | 0.51 |  | 0.10 |  |
| F (df = 3, 125) | 45.44 | < .001 | 5.78 | < .001 |

**^a^Values are standardised regression coefficients. Regression analyses used listwise deletion.**
