# Supplementary material for: AI Perceptions, Professional Identity, and AI-Supported Clinical Decisions Among Medical Students and Clinicians: Cross-Sectional Survey and Quasi-Randomized Vignette Study
Source: JMIR Med Educ. 2026 Sep 30;12:e104151. doi: 10.2196/104151 (PMC13669706; doi:10.2196/104151)
Supplement: Supplementary file 6 [file mededu_v12i1e104151_app6.docx]

Additional Phase 2 Analyses and Vignette Group Characteristics

Table S1. Significant exploratory Spearman correlations between standardised dimension items and vignette-specific contextual items by scenario

| **Scenario** | **Dimension** | **Dimension item (short)** | **Vignette-specific item (short)** | **ρ** | **P value** | **N** |
| --- | --- | --- | --- | --- | --- | --- |
| Watson | Explainability | Safer implementing AI when I can follow its reasoning | Accept AI only if guideline-conform | −0.66 | <.001 | 24 |
| Watson | Explainability | Lack of transparency makes acceptance difficult | Hard to trust AI when basis is not explainable | 0.74 | <.001 | 24 |
| Watson | Explainability | Important to fully understand AI reasoning | More likely to follow AI if it explains its process | 0.51 | .011 | 24 |
| Triage | Trustworthiness | Scientific basis & data strengthen my trust | AI helps quick decisions under pressure | 0.68 | <.001 | 21 |
| Triage | Trustworthiness | Colleagues’ trust influences my own | Trust AI more if colleagues report positive experiences | 0.44 | .045 | 21 |
| Triage | Responsibility | Feel obliged to critically review AI recommendations | Clinical experience gives me more confidence than AI | 0.63 | .002 | 21 |
| OncoGuide | Trustworthiness | Colleagues’ trust influences my own | If patient understands AI, I am more likely to follow it | 0.62 | .001 | 23 |
| OncoGuide | Explainability | Important to fully understand AI reasoning | If patient understands AI, I am more likely to follow it | −0.46 | .026 | 23 |

Table S2. Professional characteristics by vignette scenario in Phase 2

| **Characteristic** | **Watson (n=24), n (%)** | **Triage (n=21), n (%)** | **OncoGuide (n=23), n (%)** | **Total (N=68), n (%)** | **P value^a^** |
| --- | --- | --- | --- | --- | --- |
| **Clinical specialty** |  |  |  |  | .46 |
| General Practice and Internal Medicine | 2 (8.3) | 3 (14.3) | 4 (17.4) | 9 (13.2) |  |
| Surgery and Operative Specialties | 11 (45.8) | 5 (23.8) | 6 (26.1) | 22 (32.4) |  |
| Anaesthesiology and Intensive Care Medicine | 2 (8.3) | 0 (0.0) | 2 (8.7) | 4 (5.9) |  |
| Radiology and Imaging Specialties | 1 (4.2) | 4 (19.0) | 3 (13.0) | 8 (11.8) |  |
| Neurosciences and Mental Health | 2 (8.3) | 1 (4.8) | 2 (8.7) | 5 (7.4) |  |
| Paediatrics and Adolescent Medicine | 2 (8.3) | 3 (14.3) | 0 (0.0) | 5 (7.4) |  |
| Women’s and Family Health | 1 (4.2) | 2 (9.5) | 0 (0.0) | 3 (4.4) |  |
| Other Specialty | 3 (12.5) | 3 (14.3) | 6 (26.1) | 12 (17.6) |  |
| **Experience with AI in medicine** |  |  |  |  | .87 |
| Yes, regularly | 7 (29.2) | 4 (19.0) | 4 (17.4) | 15 (22.1) |  |
| Yes, occasionally | 9 (37.5) | 11 (52.4) | 14 (60.9) | 34 (50.0) |  |
| No, but I have theoretical knowledge about it | 5 (20.8) | 4 (19.0) | 5 (21.7) | 14 (20.6) |  |
| No, I have little or no experience with AI | 3 (12.5) | 2 (9.5) | 0 (0.0) | 5 (7.4) |  |

^a^ P values are based on Fisher-Freeman-Halton exact tests with Monte Carlo estimation.
